# Systems Science for Caribbean Health: the development and piloting of a model for guiding policy on diabetes in the Caribbean

**DOI:** 10.1186/s12961-016-0150-z

**Published:** 2016-10-26

**Authors:** L. Guariguata, C. Guell, T. A. Samuels, E. A. J. A. Rouwette, J. Woodcock, I. R. Hambleton, N. Unwin

**Affiliations:** 1 University of the West Indies, Cave Hill, Barbados; 2 MRC Epidemiology Unit, University of Cambridge, Cambridge, United Kingdom; 3 Nijmegen School of Management, Radboud University, Nijmegen, The Netherlands

**Keywords:** System dynamics modelling, Health policy, Policy simulation, Non-communicable disease, Developing countries, Diabetes

## Abstract

**Background:**

Diabetes is highly prevalent in the Caribbean, associated with a high morbidity and mortality and is a recognised threat to economic and social development. Heads of Government in the Caribbean Community came together in 2007 and declared their commitment to reducing the burden of non-communicable diseases (NCDs), including diabetes, by calling for a multi-sectoral, systemic response. To facilitate the development of effective policies, policymakers are being engaged in the development and use of a system dynamics (SD) model of diabetes for Caribbean countries.

**Methods:**

Previous work on a diabetes SD model from the United States of America (USA) is being adapted to a local context for three countries in the region using input from stakeholders, a review of existing qualitative and quantitative data, and collection of new qualitative data. Three country models will be developed using one-on-one stakeholder engagement and iterative revision. An inter-country model will also be developed following a model-building workshop. Models will be compared to each other and to the USA model. The inter-country model will be used to simulate policies identified as priorities by stakeholders and to develop targets for prevention and control. The model and model-building process will be evaluated by stakeholders and a manual developed for use in other high-burden developing regions.

**Discussion:**

SD has been applied with success for health policy development in high-income country settings. The utility of SD in developing countries as an aid to policy decision-making related to NCDs has not been tested. This study represents the first of its kind.

## Background

Diabetes, the vast majority of which is type 2 diabetes, is estimated to affect over 8% of the global adult population, with 80% of all cases in low- and middle-income countries (LMICs) [1]. Diabetes is associated with a marked increased mortality, particularly in younger and middle-aged adults [2], and with healthcare costs that are on average two to three times higher than in age- and sex-matched individuals without diabetes [3]. In common with many other middle-income regions and countries, the Caribbean has high diabetes prevalence, estimated to affect between 10% and 20% of most adult populations [1, 4]. Age-adjusted mortality from diabetes in the Caribbean is 35% higher than in the neighbouring United States of America (USA) [5] and complication rates, where they have been studied, such as lower extremity amputation [6], are among the highest recorded rates. The prevalence of diabetes continues to rise in the Caribbean, as it does in other LMICs [1], associated with an increasing prevalence of the major risk factors for type 2 diabetes, especially overweight and obesity, and physical inactivity [7, 8]. The prevention of type 2 diabetes, and the management of people with diabetes, present major challenges to health systems in LMICs.

The threat posed to the economic and social development of the Caribbean by diabetes and related non-communicable diseases (NCDs) was recognised by the Heads of Government summit of the Caribbean Community (CARICOM) in 2007, resulting in the Port of Spain Summit Declaration on NCDs (POSD) [9]. This was a world first for intergovernmental action on NCDs in a middle-income region, and it strongly influenced the initiation and content of the United Nations High Level Meeting on NCDs in September 2011. Both the POSD and United Nations meeting outputs (including the subsequent WHO Global Action Plan [10]) strongly emphasise the importance of policy measures for reducing NCD risk factors. However, evidence on how to achieve a reduction in overweight/obesity and physical inactivity at the population level is scarce. Despite the fact that governments in CARICOM are publically and officially committed to addressing NCDs, progress on achieving the goals set forth in the POSD has been varied [11].

It is increasingly acknowledged that the determinants of obesity and physical inactivity are embedded in the organisation of modern societies [12]. Systems science offers advantages over other multilevel or ecological frameworks in identifying the most effective points to intervene [13]. System dynamics modelling has established a methodology for engaging with policymakers and other stakeholders to facilitate broader thinking on the mechanisms and effects of complex problems such as type 2 diabetes and other NCDs. This includes the use of stakeholder in-depth interviews and group model building workshops to build and refine qualitative conceptual system models and quantitative models, and can use games or ‘flight simulators’ for appraising the potential impact of different policy options as an aid to policy decision-making [14].

### System dynamics models in diabetes and NCDs

System dynamics models have been recognised for their importance and potential in public health [15] and successfully used in high-income settings, especially in the USA, in guiding policy for the prevention and control of NCDs. The Centers of Disease Control and Prevention (CDC) sponsored the development of disease models for diabetes [16] and obesity [17], as well as a national chronic disease strategy [18] that enabled simulation of the trajectories of NCDs and supported appropriate public health planning in the country. Another more locally-based model looked at health planning and system structure for NCD management and prevention [19] in Washington State. Beyond the USA, New Zealand adapted the USA Cardiovascular Disease Prevention model [20] to its context and used it for health planning, while a team in Canada developed a stratified model to look at the social determinants of disease in Toronto and its effects on underserved populations [21]. While all of these models show promise in using system dynamics methodologies for health policy and planning, none have been extended to NCDs in LMICs or engaged directly with policymakers.

The study described here is adapting and developing a system dynamics model to guide policy on the prevention and control of diabetes in a middle-income region, using a process designed to fully engage with policymakers and other multi-sectoral stakeholders, and evaluate the utility of the process and resulting simulation model from their perspective.

### Study objectives

The overarching aim of the study is to use system dynamics modelling to explain the rise in the diabetes burden for middle-income countries in the Caribbean and assist in policy decision-making through appraising the potential impact of different strategies and policies for prevention and control.

The main study phases will be to:

1. Undertake, with policymakers and other stakeholders, in-depth interviews and a quantitative data evidence review on the systems driving obesity, physical inactivity and access to effective diabetes care
2. Accordingly develop a model for use in the Caribbean, based on the conceptual models and evidence reviews
3. Use, along with policymakers, the simulation model to explore the potential impacts and interactions of different policy options, including the feasibility of achieving relevant targets from the WHO Global Monitoring Framework [22]
4. Evaluate from the perspectives of the policymakers and stakeholders the utility of both the qualitative and quantitative aspects of the modelling process

## Design

### Setting and target populations: building on the POSD evaluation study

**Fig. 1:**
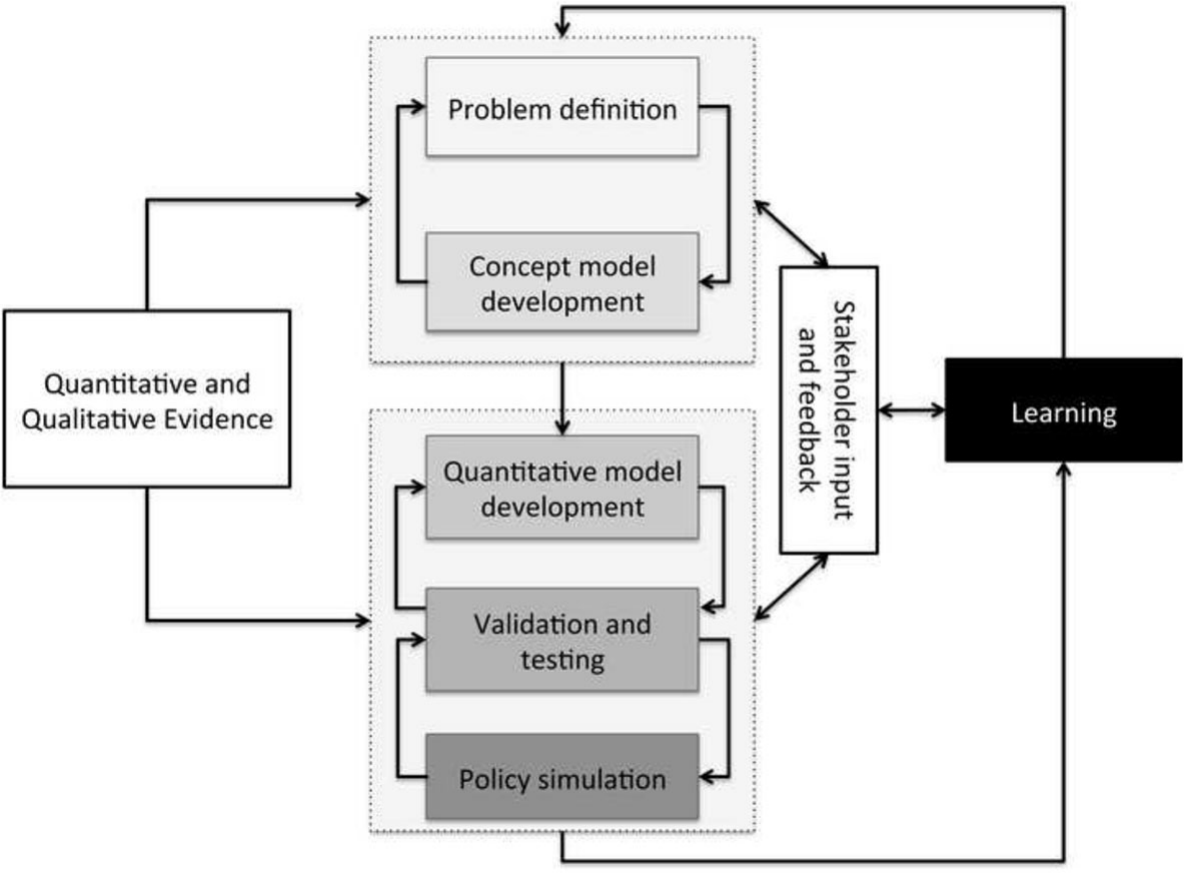
Overview of process* for system dynamics model development. *Developing a system dynamics model is not a strictly linear process though it has a progression; it is marked by iteration, revision and feedback at each point in the process

A multi-country evaluation of the impact of the 2007 POSD is currently underway (POSD evaluation study: http://www.onecaribbeanhealth.org/), led by the University of the West Indies, to assess successes and gaps in the development and implementation of policy on NCDs in the Caribbean region. The POSD evaluation study has developed a network of stakeholders and collected a range of qualitative and quantitative data that can serve directly as resources for the development of the model described here. As part of this study, data are being collated on mortality, morbidity and risk factor trends across the 20 CARICOM member states. A core component of the POSD evaluation study were case studies in seven Caribbean countries of the policy responses to NCDs, including qualitative in-depth interviews with stakeholders from government and other sectors. In addition, government and regional policy initiatives around the prevention and control of NCDs since 2007 are being appraised. The model development will build on findings and stakeholder contacts from the POSD evaluation study (Fig. 1).

Middle-income countries were chosen to evaluate system dynamics methodologies in health in resource-limited settings. One country is Jamaica, as the largest English-speaking middle-income country in the Caribbean. The second is Belize, which is politically and culturally part of the Caribbean, but has a unique environment being a Central American mainland, English-speaking middle-income country with access to data on diabetes prevalence, risk factors and its effects from continuing monitoring in the country. The third is St Vincent and the Grenadines, a small, middle-income island nation.

### Development of an initial conceptual model

**Fig. 2:**
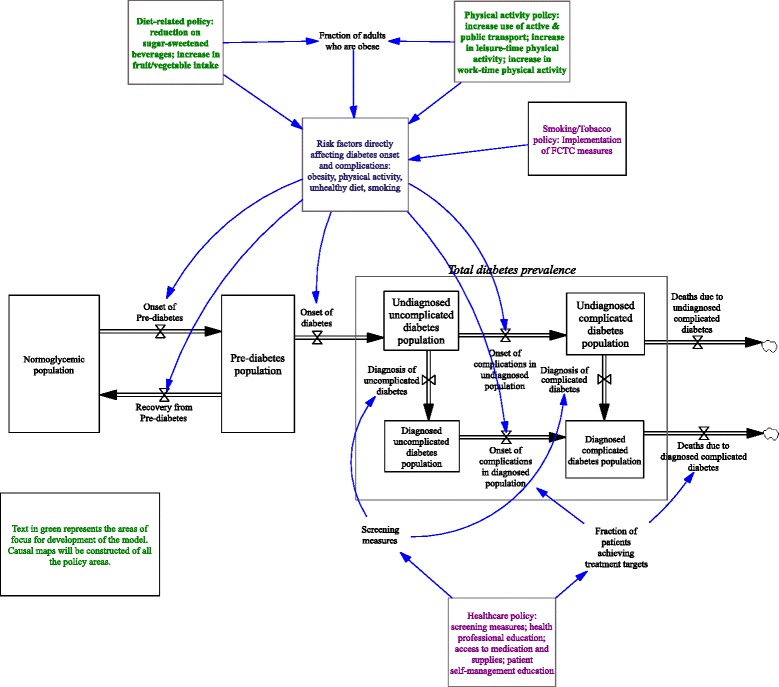
Overview of core conceptual model to be further developed with stakeholders and policymakers

Milstein et al. [23] developed a comprehensive diabetes model for the CDC in the USA to reflect, as much as possible, a generic model for diabetes. The core of this model will be used as a basis in this project. The CDC model will be adapted to a Caribbean context, which differs from the USA in demographics, ethnic admixture, health system structure, food policy, built environment and resources. Furthermore, policymakers participating in the POSD evaluation study have expressed the need to include upstream determinants of diabetes and other NCDs in any policymaking endeavour. These include the broader determinants of diet and physical activity, such as the price and availability of different foods and the structure of the built environment. The CDC model explicitly excluded these determinants as outside the model boundaries set by stakeholders partly due to a lack of sufficient evidence to satisfy stakeholders and partly because the model, as it was constructed, satisfied the purposes of that study [16]. The policies of interest for evaluation in this study also differ from those of the CDC and thus the model must follow the needs of the context for which it is being designed. Despite these differences, a core epidemiological structure exists in the CDC model, which is generic and will be used as the starting point for model adaptation and development (Fig. 2).

This initial concept model will be used to engage stakeholders and promote systems thinking during in-depth interviews. The model focuses explicitly on incorporating shared risk factors for NCDs and their upstream determinants, which are potential targets of policy interventions. Total diabetes prevalence will be used as the primary outcome measure of interest (in system dynamics referred to as the ‘reference mode’) for policy interventions.

The interviews will be used to gather qualitative data from stakeholders on their perspectives regarding diabetes, its determinants, and effects in their own countries and the region. The development will be an iterative process with each phase informing the other, thus refining the model. As part of this iterative process, the model structure will be compared to the CDC diabetes model [23]. This will provide an additional way of promoting critical consideration of the model structure through understanding the basis and the consequences of any differences.

### Review of existing qualitative data and stakeholder selection

The POSD evaluation study conducted in-depth interviews with NCD experts in countries on their views regarding progress on policies laid out by the POSD. Using data collected from the evaluation the investigators will undertake a formal thematic qualitative analysis of existing interview data as it relates to drivers and determinants of the diabetes system in the region, particularly to the upstream determinants of diet and physical activity through obesity. The review will also be used to identify stakeholders to be invited to participate in and for follow-up in-depth interviews, and insights from the prior interviews will be revisited and discussed in these planned follow-ups. Further stakeholders will be identified using snowball sampling and referral from contacts within and using the existing POSD network in the three project countries. For practical reasons, and in keeping with recommendations for model building [24], no more than 10 stakeholders will be selected for each country.

### Review of quantitative evidence

A systematic literature review will provide evidence on the quantitative historical relationships between variables in the model. The empirical data will be used to calibrate the model. A systematic review is being conducted of studies performed within the last 10 years in the Caribbean describing the prevalence of type 2 diabetes, its risk factors and their social determinants, and healthcare coverage and outcomes in people with diabetes. This evidence review will build upon recent scoping and systematic reviews into the social determinants of diabetes in the Caribbean [25]. In addition, any available, unpublished, datasets in the three project countries will be identified. This will be facilitated by the POSD evaluation and ongoing work collating mortality and risk factor datasets within the CARICOM countries. In addition, stakeholders will be asked during the qualitative interviews, if they know of any local datasets.

In the absence of sufficient quantitative data from the Caribbean for a relationship within the conceptual model, it will be necessary to use data for calibration from other parts of the world. Care will be taken to use data matching a context and ethnic mix as close as possible to the Caribbean populations and such data will be assumed to come with greater uncertainty in the calibration exercise.

### Stakeholder interviews and new data collection

Stakeholders for the in-depth qualitative interviews will involve members of the Ministries of Health, including the Chief Medical Officer or NCD focal point, other relevant government ministries, such as education, transport and agriculture; leaders from civil society organisations, including diabetes organisations and church groups; and prominent private sector leaders.

A semi-structured interview guide will be used, eliciting stakeholders’ estimation and gaining feedback on the draft preliminary model, and contextual information of trends in diabetes, obesity and physical inactivity, and the underlying determinants in their settings; similarly, stakeholder views on trends in access to diabetes care and determinants of blood glucose, pressure, and lipid control and their determinants will be explored. All interviews will be recorded, transcribed verbatim and analyzed thematically [26], and used to generate causal maps [27].

### Country-level model building

A conceptual model will be developed for each of the three study countries using stakeholder input and data gathered from the evidence review. Stakeholders will be given a report of the conceptual model and given an opportunity to submit changes for one round of iteration. The models will be finalised and a quantitative model produced for each country using country-level data where possible. The models will be compared and simulations will be produced using a single set of policies and scenarios for testing.

### Group model building and an inter-country regional model

The investigators will use a revised conceptual model to include input from country-level work as the basis for the development of an inter-country regional model. The model development will include stakeholders with regional expertise who will meet for a group model building workshop. Over 2 days, the workshop will provide an introduction to system dynamics modelling and systems thinking to stakeholders to develop a regional diabetes model. Following the model building workshop, stakeholders will be given two opportunities for revision and feedback. These comments will be included in the revision and development of the model. Once there is general agreement from stakeholders on the conceptual model, evidence from quantitative data collection will be applied to develop a quantitative model.

### Quantitative inter-country regional model, validation and policy simulations

Once the conceptual model has been finalised, estimates for each of the parameters and variables will be incorporated using data obtained from the quantitative data collection. The model will be used to try and replicate trends in risk factors and outcomes available from studies in the region as much as possible from 2000 to the present. One of the time frames that will be used for simulations is to 2025 in order to help guide potential policies for achieving relevant targets of the WHO Global Monitoring Framework [22]. The model will also be extended to 2050 to give enough time to assess the presence and influence of slow feedback mechanisms. Testing the model will involve undertaking sensitivity analyses for the parameters included and also ‘extreme case’ modelling [28] will help to assess the plausibility of the simulations being produced.

The primary targets for the option appraisal and simulation will be centred on policy recommendations generated from the POSD evaluation study, relevant targets in the Global Monitoring Framework, and policies identified by stakeholders, including no increase in obesity or diabetes prevalence by 2025, and a reduction in premature diabetes mortality by 25%. Various alternative scenarios, including adoption and implementation of policies related to risk factors and treatment, exogenous scenarios and testing one against the other, will be evaluated using the simulations. Potential policies will be evaluated alone and in plausible combinations, as guided by stakeholders, against a timeline of achievement by 2025, without a time horizon, and estimating a time horizon for achievement.

Special attention will be put into stratifying the model by gender and socioeconomic status to explore how the potential interventions for the prevention and control of diabetes impact on health equity.

### Stakeholder model evaluation

Once the quantitative model has been tested, a simulation test environment (management flight simulator) will be developed for stakeholders to interact with the model. Stakeholders will be interviewed and surveyed to gather feedback on the model building process, utilisation of the simulation tool, and how process and outputs could be made more useful to them.

### Ethical review

The study has been submitted for ethical review and approved by the University of the West Indies, Cave Hill/Barbados Ministry of Health Research Ethics Committee/Institutional Review board.

## Discussion

The model is being constructed as part of a development project with a view toward expanding in the future. One area of particular interest is the incorporation of economic and cost estimates to the simulations to better serve policymakers in understanding ‘best buys’ and the cost-benefit relationships related to the policy choices being modelled. As part of this effort, the project is looking to establish external collaborations with economists in the region and those with expertise in systems thinking.

Diabetes shares risk factors and outcomes with the other three major classes of NCDs (cardiovascular disease, chronic lung diseases and cancers). Together, these four disease categories make up 82% of all deaths due to chronic disease and the burden is especially high in LMICs [5]. It is of high public health importance to consider the burden and effects of all NCDs in the CARICOM region. As a result, any further development of the model must take into account how interventions affect the shared risk factors and outcomes of all of these diseases. In addition, in keeping with system dynamics methodology, the model will be considered as in development with further iteration, adaptation and modification encouraged for its improvement.

The model is designed to promote the engagement of policymakers in evidence-based decision-making and policy design. Collaboration between researchers and policymakers and skills-building are major determinants influencing the use of evidence for supporting policy [29]. The protocol described here makes explicit these interactions through group model building and integrates learning in systems modelling and interpretation for policymakers. Furthermore, the continued development of the model with stakeholders will provide a platform for sustained interaction and engagement in policy development and support.

The methodology used for the development of the model may also be of interest to other developing countries facing a similar diabetes burden under constrained resources. The methodology will be written up as a manual for use by other interested regions and in particular Small Island Developing States, including in the Western Pacific and Indian Ocean, faced with similar disease burdens, policy issues and resource constraints as the Caribbean region.

## Abbreviations

CARICOM: Caribbean Community
CDC: Centers for Disease Control
LMICs: low- and middle-income countries
NCDs: non-communicable diseases
POSD: Port of Spain Declaration
USA: United States of America

## References

[1] Guariguata L, Whiting DR, Beagley J, Linnenkamp U, Hambleton I, Cho NH (2014). Global estimates of diabetes prevalence in adults for 2013 and projections for 2035 for the IDF Diabetes Atlas. Diabetes Res Clin Pract.

[2] Roglic G, Unwin N (2010). Mortality attributable to diabetes: estimates for the year 2010. Diabetes Res Clin Pract.

[3] Zhang P, Zhang X, Brown J, Vistisen D, Sicree R, Shaw J (2010). Global healthcare expenditure on diabetes for 2010 and 2030. Diabetes Res Clin Pract.

[4] Yisahak SF, Beagley J, Hambleton IR, Narayan KMV (2014). Diabetes in north America and the Caribbean: an update. Diabetes Res Clin Pract.

[5] Lozano R, Naghavi M, Foreman K, Lim S, Shibuya K, Aboyans V (2012). Global and regional mortality from 235 causes of death for 20 age groups in 1990 and 2010: a systematic analysis for the Global Burden of Disease Study 2010. Lancet.

[6] Hennis AJM, Fraser HS, Jonnalagadda R, Fuller J, Chaturvedi N (2004). Explanations for the High Risk of Diabetes-Related Amputation in a Caribbean Population of Black African Descent and Potential for Prevention. Diabetes Care.

[7] Ng M, Fleming T, Robinson M, Thomson B, Graetz N, Margono C (2014). Global, regional, and national prevalence of overweight and obesity in children and adults during 1980–2013: a systematic analysis for the Global Burden of Disease Study 2013. Lancet.

[8] Hallal PC, Andersen LB, Bull FC, Guthold R, Haskell W, Ekelund U (2012). Global physical activity levels: surveillance progress, pitfalls, and prospects. Lancet.

[9] Declaration of Port-of-Spain : Uniting to stop the Epidemic of Chronic NCDs. 2007. http://caricom.org/media-center/communications/statements-from-caricom-meetings/declaration-of-port-of-spain-uniting-tostop-the-epidemic-of-chronic-ncds. Accessed 11 Mar 2016.

[10] World Health Organization (2013). Global action plan for the prevention and control of noncommunicable diseases 2013–2020.

[11] Samuels TA, Kirton J, Guebert J (2014). Monitoring compliance with high-level commitments in health: the case of the CARICOM Summit on Chronic Non-Communicable Diseases. Bull World Health Organ.

[12] Stuckler D, Siegel K (2011). Sick Societies: Responding to the global challenge of chronic disease.

[13] Kumanyika SK, Parker L, Sim LJ (2010). Committee on an Evidence Framework for Obesity Prevention Decision Making; Food and Nutrition Board; Institute of Medicine. Bridging the Evidence Gap in Obesity Prevention: A Framework to Inform Decision Making.

[14] Vennix JAM (1996). Group Model Building: Facilitating Team Learning Using System Dynamics.

[15] Homer JB, Hirsch GB (2006). System dynamics modeling for public health: background and opportunities. Am J Public Health.

[16] Jones AP, Homer JB, Murphy DL, Essien JDK, Milstein B, Seville DA (2006). Understanding diabetes population dynamics through simulation modeling and experimentation. Am J Public Health.

[17] Homer J, Milstein B, Dietz W, Buchner D, Majestic E. Obesity population dynamics: exploring historical growth and plausible futures in the US. http://www2.cdc.gov/syndemics/pdfs/Obesity%20population%20dynamics%20(Homer,%20ISDC-06).pdf. Accessed 11 Mar 2016.

[18] Homer J, Milstein B, Wile K, Trogdon J, Huang P, Labarthe D (2010). Simulating and evaluating local interventions to improve cardiovascular health. Prev Chronic Dis.

[19] Hirsch G, Homer J, Evans E, Zielinski A (2010). A System Dynamics Model for Planning Cardiovascular Disease Interventions. Am J Public Health.

[20] Kenealy T, Rees D, Sheridan N, Moffitt A, Tibby S, Homer J (2012). A “whole of system” approach to compare options for CVD interventions in Counties Manukau. Aust N Z J Public Health.

[21] Mahamoud A, Roche B, Homer J (2013). Modelling the social determinants of health and simulating short-term and long-term intervention impacts for the city of Toronto, Canada. Soc Sci Med.

[22] World Health Organization. Draft comprehensive global monitoring framework and targets for the prevention and control of noncommunicable diseases. Geneva: WHO; 2013. (Sixty-sixth World Health Assembly). Report No.: A66/8.

[23] Milstein B, Jones A, Homer JB, Murphy D, Essien J, Seville D (2007). Charting plausible futures for diabetes prevalence in the United States: a role for system dynamics simulation modeling. Prev Chronic Dis.

[24] Hovmand P (2013). Community Based System Dynamics.

[25] Sobers-Grannum N, Murphy MM, Nielsen A, Guell C, Samuels TA, Bishop L (2015). Female gender is a social determinant of diabetes in the Caribbean: a systematic review and meta-analysis. PLoS One.

[26] Green J, Thorogood N (2004). Qualitative Methods for Health Research.

[27] Kim H, Andersen DF (2012). Building confidence in causal maps generated from purposive text data: mapping transcripts of the Federal Reserve. Syst Dyn Rev.

[28] Barlas Y (1994). Model validation in system dynamics. Proceedings of the 1994 International System Dynamics Conference.

[29] Oliver K, Innvar S, Lorenc T, Woodman J, Thomas J (2014). A systematic review of barriers to and facilitators of the use of evidence by policymakers. BMC Health Serv Res.

